# Disseminated chickenpox with multi-organ failure in an immunocompetent adult. a case report and literature review

**DOI:** 10.1186/s12879-025-11015-0

**Published:** 2025-05-20

**Authors:** Jaweria Akram, Abdul Qadir, Hadeel Alfar, Theresa Paul, Mamunul Islam, Ali Rahil

**Affiliations:** 1 Department of Medicine, Hamad Medical Corporation, HGH, P.O. Box 3050, Doha, Qatar; 2 Cook County Health, Chicago, IL USA; 3 Medical Education Department, Hamad Medical Corporation, Doha, Qatar

**Keywords:** Coagulopathy, Disseminated chickenpox infection, Fulminant hepatitis, Varicella zoster in immune-competent adults

## Abstract

**Background:**

Disseminated varicella infection in adults, particularly those with mild immunosuppression, can lead to severe complications and life-threatening outcomes. Early diagnosis and intervention are critical yet challenging due to atypical presentations.

**Case presentation:**

A previously healthy 26-year-old male presented with severe epigastric and right upper quadrant abdominal pain radiating to the back, nausea, loss of appetite, and constipation. He had been taking oral prednisolone 5 mg daily for 2 weeks prior to symptom onset. Initial evaluation showed mild liver enzyme elevation but normal imaging studies. Within two days, he developed widespread vesicular lesions consistent with varicella, rapidly progressing to fulminant hepatic failure, disseminated intravascular coagulation (DIC), acute renal failure, and acute respiratory distress syndrome (ARDS). Despite intensive management including antiviral therapy, antibiotics, supportive transfusions, and mechanical ventilation, the patient unfortunately passed away due to multi-organ failure.

**Conclusion:**

This case emphasizes the importance of early suspicion and diagnosis of varicella infection in adults presenting with atypical symptoms, even low-dose steroid therapy may cause sufficient immunosuppression to predispose individuals to disseminated viral infections. Timely intervention can significantly influence patient outcomes.

## Introduction

Varicella-zoster virus (VZV) is one of eight herpesviruses known to cause human infection. VZV infection causes two clinically distinct forms of disease: varicella (chickenpox) and herpes zoster (shingles). Primary VZV infection results in the diffuse vesicular rash of varicella, or chickenpox. Endogenous reactivation of latent VZV typically results in a localized skin infection known as herpes zoster, or shingles.

Primary varicella infection in children is generally a mild disease compared to more severe presentations in adults or immunocompromised patients of any age. However, in adults, particularly those immunocompromised or even mildly immunosuppressed, varicella infection can manifest as severe, disseminated disease leading to life-threatening complications. Prompt recognition of atypical presentations is critical in improving outcomes. Here, we describe an unusual and severe case of disseminated varicella infection complicated by multi-organ failure in a previously healthy young adult, highlighting the importance of clinical vigilance and early intervention.

## Case presentation

A previously healthy 26-year-old male presented to the emergency department (ED) with a 2-day history of severe, colicky epigastric and right upper quadrant (RUQ) abdominal pain radiating to the back, rated 9/10 in severity, accompanied by nausea, loss of appetite, and constipation. He was taking prednisolone 5 mg daily, ibuprofen 400 mg three times daily, and gabapentin 200 mg daily for two weeks, prescribed by a primary care physician for presumed neuropathic back pain, which was described as burning in character and radiating to the lower limbs. He was not evaluated in a hospital setting for his back pain. The patient denied fever, chills, or jaundice. He reported contact with a co-worker who was diagnosed with chickenpox four days prior to symptom onset. He received all childhood immunization, but he was not immunized against chicken pox.

On initial assessment, the patient was alert and oriented, with stable vital signs (BP: 118/72 mmHg, HR: 72 bpm, RR: 19 breaths/min, Temp: 36.8 °C, SpO2: 100%). Examination revealed significant tenderness localized to the epigastric region without guarding or rebound tenderness. Laboratory results at presentation were largely within normal limits except mildly elevated liver enzymes (ALT 46 U/L, AST 54 U/L) (Table 1).

**Fig. 1:**
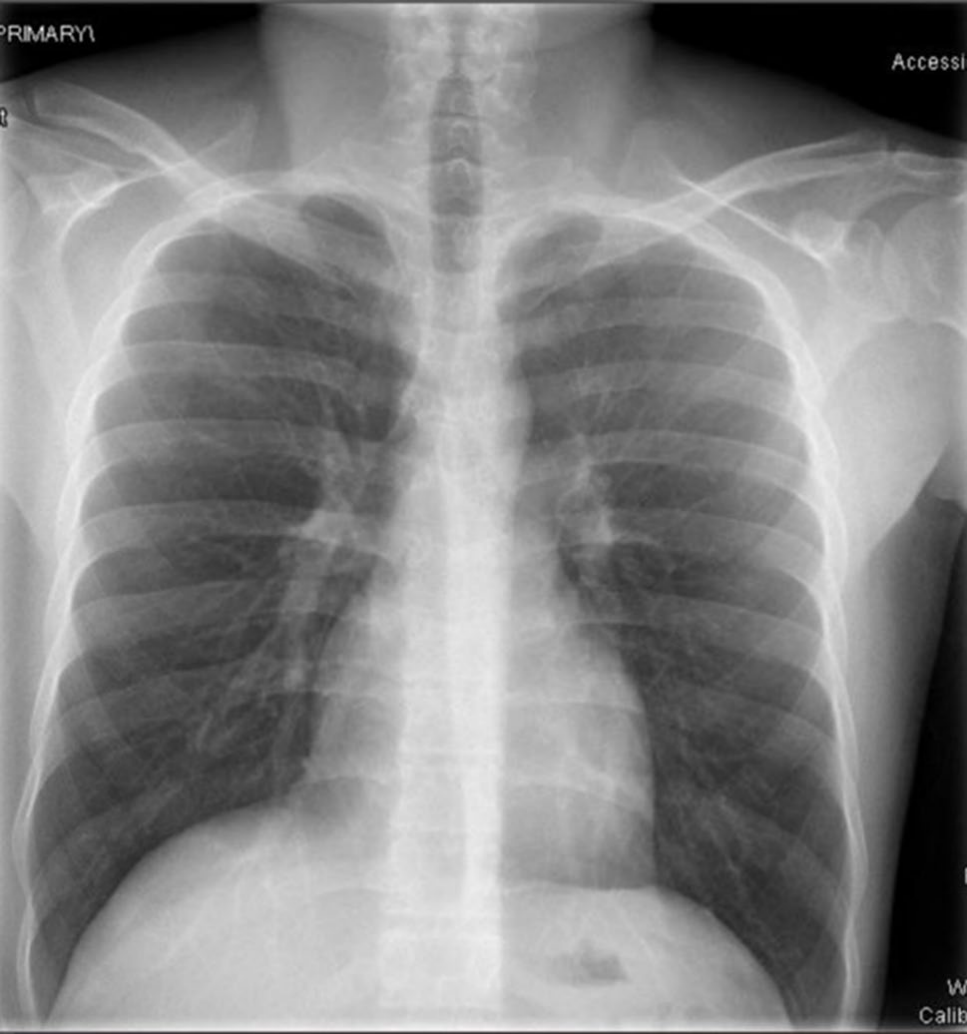
Day 1 Chest XR: AP view showing bilateral diffuse alveolar opacities with increased reticular shadows suggestive of ARDS, normal cardiac shadow, NGT and central line are in situ

Initial imaging, including ultrasound and contrast-enhanced CT of the abdomen, was unremarkable, ruling out acute cholecystitis, biliary pathology, and intestinal ischemia. Initial chest X-ray was also normal (Fig. 1). Pantoprazole 40 mg IV once daily was started for stress ulcer prophylaxis. Despite analgesic therapy including paracetamol (1 g IV), morphine (10 mg/ml), and fentanyl (40 mcg), abdominal pain persisted and worsened, eventually accompanied by severe back pain. The patient received six grams of paracetamol over 2 days (three doses/day). While this dose is within the upper therapeutic limit, it may contribute to liver injury in a compromised liver.

On day 2 of admission, the patient developed widespread vesicular eruptions beginning on his forehead and spreading caudally, sparing the palms and soles, consistent with varicella infection. Concomitantly, his clinical condition rapidly deteriorated. Follow-up laboratory tests showed severe hepatic dysfunction (ALT 1437 U/L, AST > 1000 U/L), profound coagulopathy (INR 3.5, markedly prolonged PT/APTT, fibrinogen < 0.3 gm/L), leukocytosis (WBC 23.6 × 10³/µL), and elevated inflammatory markers (CRP 15.0 mg/L, Procalcitonin 0.12 ng/mL). Varicella polymerase chain reaction (PCR) from vesicular lesions was positive, confirming disseminated varicella zoster infection.

His condition further complicated by hemorrhagic transformation of vesicles, subconjunctival hemorrhage, active bleeding from intravenous sites, and progressive encephalopathy. The patient developed altered mental status with decreased Glasgow Coma Score (GCS), prompting evaluation for hepatic encephalopathy. As a result, investigations were undertaken to exclude other potential causes of acute liver failure, including viral, autoimmune, and drug-induced etiologies. Empiric intravenous acyclovir therapy, broad-spectrum antibiotics (piperacillin-tazobactam), supportive transfusions (fresh frozen plasma (FFP), cryoprecipitate), and intensive care management were promptly initiated.

**Fig. 2:**
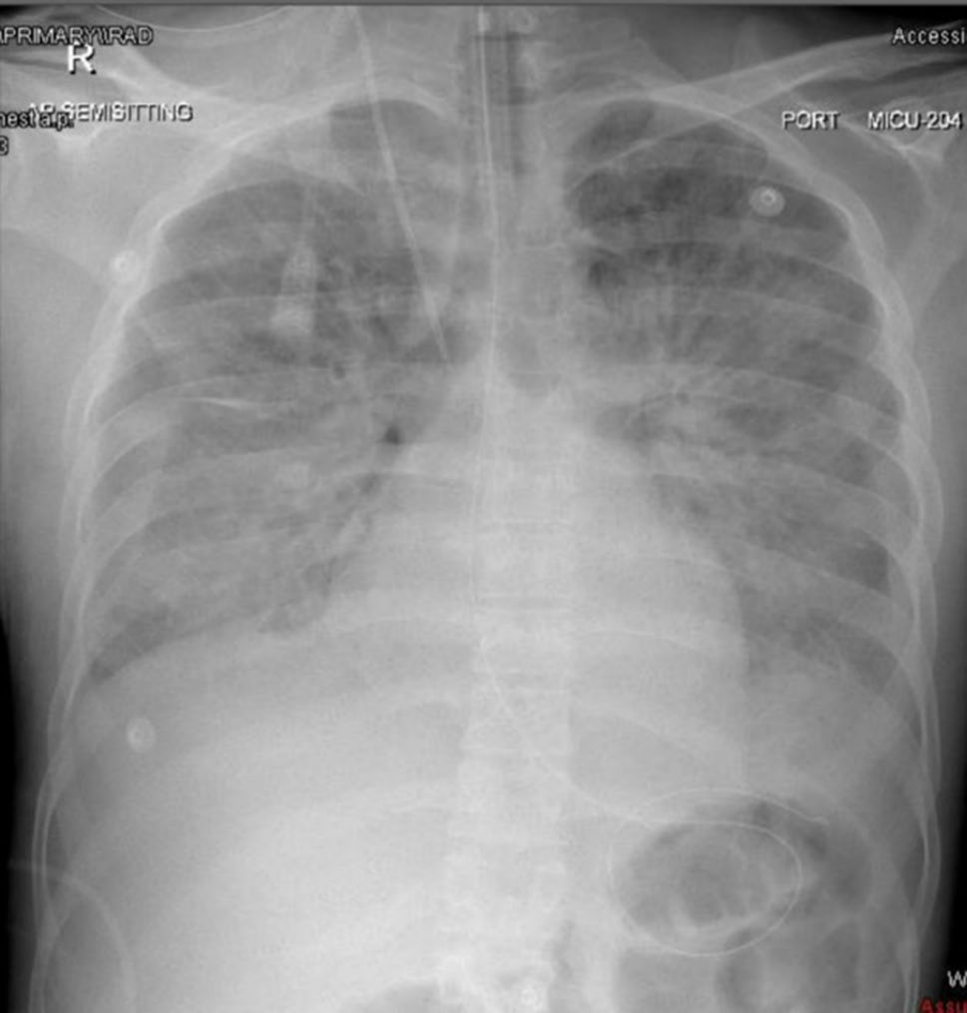
Day 3. Chest XR: AP view showing bilateral diffuse alveolar opacities with increased reticular shadows suggestive of ARDS, normal cardiac shadow, NGT and central line are in situ

The patient met clinical and laboratory criteria for disseminated intravascular coagulation (DIC), including prolonged PT/aPTT, elevated INR (4.9), low fibrinogen (< 0.3 g/L), and severe thrombocytopenia (platelets: 23 × 10^9/L), along with diffuse bleeding from cannula sites, gums, and conjunctiva. Despite maximal supportive interventions including mechanical ventilation, renal replacement therapy for acute renal failure, and vasopressor support for refractory shock, the patient’s condition progressed rapidly to multiorgan failure, DIC, and acute respiratory distress syndrome (ARDS) (Fig. 2). The patient unfortunately passed away due to cardiac arrest and multi-organ failure on the sixth day of hospitalization.

This case underscores the critical importance of early recognition of atypical presentations of varicella in adults, particularly when complicated by even low-dose steroid-induced immunosuppression, to prevent catastrophic outcomes.

**Table 1:** Shows initial laboratory investigation results.

	DAY1	DAY3	DAY4
**Hematology**:			
WBC	9.5	1128	6.4
Hgb	15.4	10.9	3.6
hct	44.9	46.6	3.6
MCV/MCH	Normal	Normal	Normal
Platelets	220	131	23
Absolute reticulocyte count	-	-	0.3
Reticulocyte index	-	-	O.11
**Coagulation profile:**			
	DAY1	DAY3	DAY4
PT (sec)	-	40.2	26.9
INR	-	3.54.9	2.7
D-dimer	-	Unable to analyze by the machine	Unable to analyze by the machine
Fibrinogen (gm/l)	-	<0.3	1.18
aPTT (sec)	-	71.7	>180
**Chemistry:**			
	Day1	Day2	Day3
Urea (mmol/l)	2.5	5	4.3
Creatinine (umol/l)	47	171	192
Na (mmol/l)	142	128	138
K (mmol/l)	3.5	4.8	5
Cl (mmol/l)	104	90	91
Hco3 (mmol/l)	26	25	13
Phosphorous (mmol/l(	-	1.9	2.5
ALT (U/L)	46	1,4372,020	1,239
AST (U/L)	54	1,0006,045	2,651
CK (mmol/l)	-	2,821	
Ammonia (umol/l)	-	-	66
Lipase (U/L)	17	-	-
Ceruloplasmin (mg/dl)	-	10	-
CRP (mg/l)	1	15	3.5
Lactic acid (mmol/l)	1.8	2.4	1.7
Procalcitonin (mg/l)	-	0.12	10
**Endocrinology:**			
	DAY1	DAY3	DAY4
Ferritin (ug/l)	-	-	64,653
**Toxicology:**			
	DAY1	DAY3	DAY4
Acetaminophen level (umol/l)	-	11.4	-
Autoimmune disease:			
	DAY1	DAY3	DAY4
ASM Ab	-	negative	-
ALKM	-	negative	-
ANA CTD int	-	negative	-
C3 (mg/l)	-	88.9	72
C4 (mg/l)	-	14.3	14.1
**Serology:**			
	DAY1	DAY3	DAY4
Varicella Zoster	-	IgM / lgG negative PCR positive	IgM /IgG negative PCR positive
Measles	-	IgM negative IgG positive	-
HCV	-	IgM/IgG/PCR negative	-
HBV	-	IgM/IgG/PCR negative	-
HIV	-	IgM/IgG/PCR negative	-
EBV (IU/L)	-	PCR positive; 1,455	-

Abbreviations: WBC– White Blood Cell count, Hgb– Hemoglobin, Hct– Hematocrit, MCV/MCH– Mean Corpuscular Volume / Mean Corpuscular Hemoglobin, PT sec– Prothrombin Time (in seconds), INR– International Normalized Ratio, aPTT– Activated Partial Thromboplastin Time, Na– Sodium, K– Potassium, Cl– Chloride, HCO₃– Bicarbonate, ALT– Alanine Aminotransferase, AST– Aspartate Aminotransferase, CK– Creatine Kinase, CRP– C-Reactive Protein, ASM Ab– Anti-Smooth Muscle Antibody, ALKM– Anti-Liver-Kidney Microsomal Antibody, ANA– Antinuclear Antibodies, HCV– Hepatitis C Virus, HBV– Hepatitis B Virus, HIV– Human Immunodeficiency Virus, EBV– Epstein–Barr Virus

### PCR for varicella from skin swab was positive

## Discussion

Diagnosis of VZV is often clinical, based on characteristic skin lesions of vesicular exanthem and associated systemic symptoms. However, diagnosis may be challenging in cases presenting with atypical features such as isolated abdominal or back pain, which can precede the rash. Such prodromal symptoms have been documented in children [1] and immunocompromised individuals, and in a limited number of reports involving immunocompetent adults as well [2–11]. These manifestations are thought to result from VZV-related myeloradiculopathy, a recognized neurological complication of the infection [12]. The diagnosis of fulminant hepatitis was based on established clinical criteria—acute onset liver dysfunction with an INR > 1.5 and hepatic encephalopathy in a patient without preexisting liver disease.

Definitive diagnosis of VZV can be made using PCR for viral DNA, serology, or direct fluorescent antibody testing. Tzanck smear may show multinucleated giant cells with intranuclear inclusions. Our case demonstrates the diagnostic challenge in recognizing disseminated varicella in its early phase, where initial symptoms mimic non-infectious gastrointestinal or neurologic pathology. Despite early hospital presentation, diagnosis was delayed due to absence of rash and normal imaging. Once the vesicular rash appeared, the disease course progressed rapidly, leading to multiorgan failure. The benefits in this case included early initiation of empirical therpy, with acyclovir, supportive ICU care, and transfusions. However, limitations included delayed consideration of VZV in differential diagnoses, and the use of immunosuppressant (prednisolone 5 mg/day) for nonspecific back pain, potentially exacerbating the viral dissemination.

The differential diagnosis of VZV infection includes smallpox, coxsackievirus, echovirus, rickettsia pox, and atypical measles. Other important considerations are meningococcemia, disseminated gonococcal infection, bullous vasculitis, infectious endocarditis, acute generalized exanthematous pustulosis, pustular psoriasis, and Mucha-Habermann disease.

Disseminated varicella can lead to serious complications, including pneumonia, encephalitis, hepatitis, and hemorrhagic manifestations. Individuals with hematologic or solid organ malignancies, diabetes mellitus, or inherited thrombophilias (such as protein C and S deficiencies) are considered at higher risk for severe disease (13–14). Complications are significantly more common in adults than in children [15]. Notably, up to 90% of varicella pneumonia cases occur in adults. Less frequent but severe complications include myocarditis, gangrene, retinitis, glomerulonephritis, Reye syndrome, Guillain-Barré syndrome (GBS), acute cerebellar ataxia, and disseminated intravascular coagulation (DIC). Patients should be placed under droplet and contact precautions until all vesicular lesions have crusted. Antiviral therapy with intravenous acyclovir should be initiated as early as possible, ideally within four days of rash onset, to reduce morbidity and mortality [16].

We reviewed the literature for reported cases of disseminated varicella-zoster virus (VZV) infection in immunocompetent adults presenting with fulminant hepatic failure and/or abdominal pain as a primary symptom. Our search, limited to English-language publications, identified approximately 10 individual case reports; no case series or systematic reviews were found (Table 2). Most patients were reported to be previously healthy without known immunosuppression. However, two patients had comorbidities potentially impacting immune response: one had hepatitis B (case 6), and another had immune thrombocytopenic purpura post-splenectomy (case 10). No cases involved known malignancy, HIV, or long-term immunosuppressive therapy. We performed a structured literature review using PubMed, Scopus, and Google Scholar databases. All the patients had abdominal pain with or without fever at time of presentation. Abdominal and back pain, though non-specific, have been documented in varicella cases as potential early manifestations. These symptoms are hypothesized to result from VZV-induced myeloradiculopathy or visceral neuropathy. The patient’s right upper quadrant pain could also be attributed to hepatic inflammation and capsular distension during the early stages of viral hepatitis. Although drug-induced liver injury cannot be entirely excluded, low acetaminophen levels (11.4 µmol/L) and lack of temporal correlation argue against it as the primary cause. All patients received treatment with acyclovir. Hepatic dysfunction was observed in every case, with six patients progressing to fulminant hepatitis. The diagnosis of fulminant hepatitis was based on established clinical criteria—acute onset liver dysfunction with an INR > 1.5 and hepatic encephalopathy in a patient without preexisting liver disease [6]. Additional complications included DIC, rhabdomyolysis, encephalitis, nephritis, thrombocytopenia, refractory distributive shock, and multiorgan failure involving both respiratory and renal systems. Despite aggressive treatment, five patients died.

**Table 2:** Summary of Literature Review.

No	Patient characteristics	Co-morbidities	Presentation	Treatment	Outcome
1 (2)	22 years old female Positive sick contact	No comorbidities, immunocompetent	Back pain radiating to back, fever, then rash appeared	Acyclovir and supportive treatment	Hepatitis, pneumonia, DIC Recovered
2 (3)	60 years old female Positive sick contact	No comorbidities; immunocompetent	Epigastric pain, vomiting, fever, rash appeared two days later	Acyclovir and supportive treatment	Hepatic failure, DIC, pneumonia, multiorgan failure Passed away
3 (4)	32 years old Positive sick contact	No comorbidities; immunocompetent	abdominal pain, fever, vomiting, rash	Acyclovir and supportive treatment	Varicella hepatitis, DIC, nephritis Passed away
4 (5)	26 years old female Positive sick contact	No comorbidities; immunocompetent	Epigastric pain, fever, rash	Acyclovir and supportive treatment	Hepatic failure, DIC. Passed away Autopsy proven multiorgan involvement
5 (6)	20 years old male	known asthmatic (Not on steroids)	Fever, abdominal and back pain, rash	Acyclovir and supportive treatment	Hepatic dysfunction, multiorgan failure, Passed away
6 (7)	40 years old male Positive sick contact	Hepatitis B (not on medications)	Rash, fever, abdominal pain, jaundice	Acyclovir and supportive treatment	Hepatic failure, renal failure, DIC, passed away due to multi organ failure
7 (8)	18 years old male	No comorbidities; immunocompetent	Abdominal pain, fever, active bleeding from nose and gums, blood in urine and stool Later developed restlessness and agitation	Acyclovir and supportive treatment	Hepatitis dysfunction, DIC, encephalitis, septicemia Recovered
8 (9)	23 years old male	No comorbidities; immunocompetent	Epigastric pain, later developed rash	Acyclovir and supportive treatment	ARDS, rhabdomyolysis, acute hepatitis, DIC Recovered
9 (10)	28 years old male	No comorbidities; immunocompetent	Rash, fever	Acyclovir and supportive treatment	Hepatitis, pneumonia, rhabdomyolysis, DIC
10 (11)	36 years female	Immune thrombocytopenia (ITP) post splenectomy	abdominal pain, nausea, fever, sore throat, vesicular rash	Acyclovir and supportive treatment	Hepatitis Recovered

Our case closely parallels those previously reported, with an initial presentation of acute abdominal pain in the absence of rash. The rash appeared later, coinciding with worsening liver function, and ultimately progressed to fulminant hepatic failure with multiorgan involvement. Our patient had a history of taking low-dose prednisolone (5 mg daily) for 2 weeks prior to the onset of symptoms. Previous reports of fulminant varicella have described similar outcomes in patients who were taking low-dose steroids, although not all studies provided exact dosages [15, 16].

## Conclusion

Although chickenpox is uncommon in adults, it often follows a more severe course with potentially life-threatening complications. This case highlights the importance of considering VZV infection in adults presenting with atypical symptoms such as isolated abdominal or back pain, especially when no clear surgical cause is found. Clinicians must maintain a high index of suspicion for varicella in adults with non-specific prodromal symptoms, as misdiagnosis and delay in treatment can be fatal. Even low-dose steroid therapy may cause sufficient immunosuppression to predispose individuals to disseminated viral infections, including fulminant varicella. This case highlights the importance of considering such risks when prescribing corticosteroids.

## Data Availability

The data that supports the finding of this case report are contained within the article. Additional information are available from the corresponding author upon reasonable request and with the approval of relevant ethics committee.
